# Bi-allelic variants in *COQ8B*, a gene involved in the biosynthesis of coenzyme Q10, lead to non-syndromic retinitis pigmentosa

**DOI:** 10.1016/j.ajhg.2024.08.005

**Published:** 2024-09-02

**Authors:** Ana Belén Iglesias-Romero, Karolina Kaminska, Mathieu Quinodoz, Marc Folcher, Siying Lin, Gavin Arno, Joaquim Calado, Andrew R. Webster, Alexandre Moulin, Ana Berta Sousa, Luisa Coutinho-Santos, Cristina Santos, Carlo Rivolta

**Affiliations:** 1 Ophthalmic Genetics Group, Institute of Molecular and Clinical Ophthalmology Basel, 4031 Basel, Switzerland; 2 Department of Ophthalmology, Universität Basel, 4031 Basel, Switzerland; 3 Department of Genetics and Genome Biology, University of Leicester, Leicester LE1 7RH, UK; 4 National Institute of Health Research Biomedical Research Centre at Moorfields Eye Hospital and the Institute of Ophthalmology, London, UK; 5 Institute of Ophthalmology, University College London, London EC1V 9EL, UK; 6 Greenwood Genetic Center, Greenwood, SC 29646, USA; 7 ToxOmics, NOVA Medical School, Universidade Nova de Lisboa, 1169-056 Lisboa, Portugal; 8 Jules-Gonin Eye Hospital, Fondation Asile des Aveugles, University of Lausanne, 1004 Lausanne, Switzerland; 9 Department of Medical Genetics, Centro Hospitalar Universitario Lisboa Norte EPE, 1649-028 Lisboa, Portugal; 10 Laboratory of Basic Immunology, Faculdade de Medicina, Universidade de Lisboa, 1649-028 Lisboa, Portugal; 11 Instituto de Oftalmologia Dr. Gama Pinto, 1150-255 Lisboa, Portugal; 12 iNOVA4Health, Universidade NOVA de Lisboa NOVA Medical School, 1150-082 Lisboa, Portugal

**Keywords:** COQ8B, retinitis pigmentosa, coenzyme Q, Mendelian diseases, inherited retinal diseases

## Abstract

Retinitis pigmentosa (RP) is a Mendelian disease characterized by gradual loss of vision, due to the progressive degeneration of retinal cells. Genetically, it is highly heterogeneous, with pathogenic variants identified in more than 100 genes so far. Following a large-scale sequencing screening, we identified five individuals (four families) with recessive and non-syndromic RP, carrying as well bi-allelic DNA changes in *COQ8B*, a gene involved in the biosynthesis of coenzyme Q10. Specifically, we detected compound heterozygous assortments of five disease-causing variants (c.187C>T [p.Arg63Trp], c.566G>A [p.Trp189Ter], c.1156G>A [p.Asp386Asn], c.1324G>A [p.Val442Met], and c.1560G>A [p.Trp520Ter]), all segregating with disease according to a recessive pattern of inheritance. Cell-based analysis of recombinant proteins deriving from these genotypes, performed by target engagement assays, showed in all cases a significant decrease in ligand-protein interaction compared to the wild type. Our results indicate that variants in *COQ8B* lead to recessive non-syndromic RP, possibly by impairing the biosynthesis of coenzyme Q10, a key component of oxidative phosphorylation in the mitochondria.

## Graphical abstract

**Figure:**
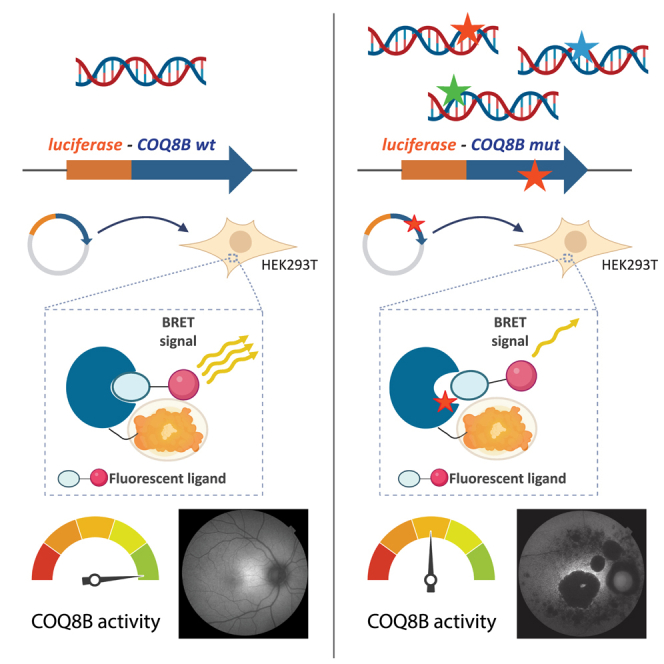

## Teaser

We identified DNA changes in a gene, *COQ8B*, that can lead to retinitis pigmentosa, a hereditary form of blindness. The product of this gene is involved in the production of coenzyme Q10, a molecule that is essential for cell energy.

## Main text

Coenzyme Q10 (CoQ10), the most common form of ubiquinone in humans, plays an essential role in mitochondrial oxidative phosphorylation and in cellular energy production.^1^ Primary coenzyme Q (CoQ) deficiencies include a collection of rare mitochondrial disorders, all with an autosomal-recessive pattern of inheritance, and due to genetic variants in elements of the CoQ biosynthetic pathway. These conditions are characterized by a very high clinical heterogeneity, likely reflecting the variable functions of the different proteins affected by disease-causing DNA changes.^2^^,^^3^^,^^4^^,^^5^^,^^6^ Primary clinical manifestations include neurologic, renal, cardiac, sensorineural, muscular, and ophthalmologic phenotypes. Neurological complications affect the central nervous system (CNS) in the form of encephalopathy. Reported manifestations include seizures, dystonia, spasticity, and/or intellectual disability.^2^ Renal involvement presents as an uncommon trait, steroid-resistant nephrotic syndrome (SRNS), which serves as a significant indicator of primary CoQ10 deficiency. Furthermore, a CoQ10 deficit can also lead to a prevalent cardiac pathology known as hypertrophic cardiomyopathy (HCM).^7^^,^^8^ Interestingly, one of the phenotypes associated with defects in proteins of the CoQ10 biosynthetic pathway is retinopathy, reported in association with variants in *PDSS1* (MIM: 607429), *COQ2* (MIM: 609825), *COQ4* (MIM: 612898), and *COQ5* (MIM: 616359) in individuals with syndromic or non-syndromic retinitis pigmentosa (RP [MIM: 268000]).^5^^,^^9^

Like other forms of inherited retinal diseases (IRDs), RP is a form of hereditary blindness characterized by the progressive death of photoreceptors, the light-sensing cells of the retina.^10^ It manifests with initial symptoms of night blindness, followed by daytime vision loss, usually occurring from the mid-periphery to the periphery and the center of the visual field.^11^ Genetically, RP is highly heterogeneous, with disease-causing variants identified in more than 100 genes (https://retnet.org/). Over the years, our understanding of the molecular genetics of RP has advanced significantly, mostly through the use of next-generation sequencing technologies. Yet, its missing heritability is still considerable, implying that a potentially high number of genes linked to IRDs still awaits molecular identification.^10^^,^^12^^,^^13^ To address this issue, we investigated the genotypes of 415 affected individuals who were negative for disease-causing variants in genes previously associated with IRDs by using genome-wide and unbiased methods, such as exome sequencing (ES) or genome sequencing (GS). An additional affected individual was identified through collaborative efforts. This genetic screening was performed in agreement with the tenets of the Declaration of Helsinki and was approved by the Ethics Committees of all our respective institutions. Written informed consent was obtained from all participating individuals prior to their inclusion in this study.

**Figure 1:**
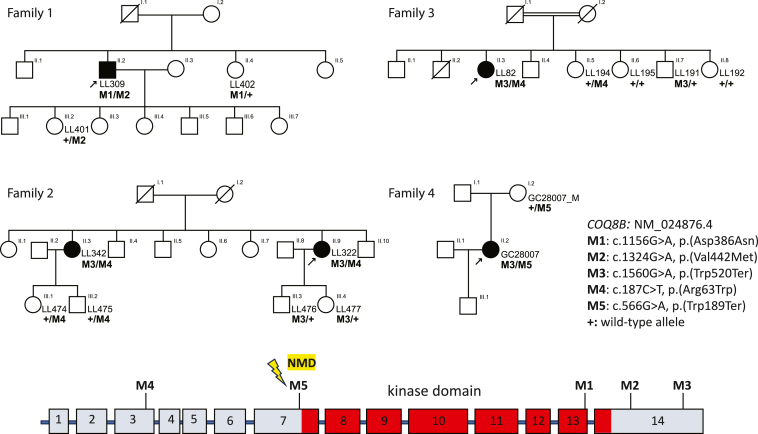
Structure of the families analyzed and their *COQ8B* genotypes Black arrows point to the proband of each family. A schematic structure of the gene (introns are not to scale) is also provided. NMD indicates the variant detected in family 4, M5, that likely triggers nonsense-mediated mRNA decay. The red area indicates the part of the coding sequence specifying the kinase domain of COQ8B.

Following this screening performed by institution-specific *in silico* pipelines developed for this purpose^14^ (see supplemental methods), we identified four families with five individuals who were found to be positive for rare and potentially disease-causing bi-allelic variants in *COQ8B* (MIM: 615567). Families 1–3 (Figure 1) were from Portugal, while one pedigree (family 4, Figure 1) was from the United Kingdom. All affected individuals were born to unaffected parents, suggesting a recessive mode of inheritance of the disease. They were all diagnosed with non-syndromic RP based on detailed ophthalmic examination, including fundoscopy and electroretinogram testing (clinical details are reported in Tables 1 and S1). LL309, LL322, LL342, and LL82 also underwent a thorough nephrological examination. None of them displayed evidence of glomerular injury: proteinuria, as assessed by the protein-creatinine ratio on a first morning urine, was distinctly absent, which rules out the diagnosis of SRNS (Table 1). They also showed non-pathological morphology/unspecific morphology findings of the kidneys, by ultrasound. Finally, all displayed normal serum creatinine.

**Table 1:** Summary clinical findings of the individuals analyzed

**Family**	**ID**	**Age at examination (years)**	**Age of onset (years)**	**Sex**	**Ocular diagnosis**	**Ocular phenotype**	**Renal phenotype**		
							**Protein-creatinine urinary ratio**^a^	**Serum creatinine**^b^	**eGFR**^c^
1	LL309	48	14	male	RP	RP with early macular involvement	15	0.91	99
2	LL322	52	30	female	RP	mild RP with annular atrophy pattern	80	0.56	126
2	LL342	64	40	female	RP	RP with spared macula	68	0.70	112
3	LL82	61	early childhood	female	RP	RP with macular edema	64	0.66	100
4	GC28007	28	26	female	RP	mild RP with macular edema	NA	NA	NA

NA, not available.

a On a first morning urine, in mg/dL. Normal reference value <200 mg/g.

b In mg/dL. Normal reference values: 0.5–1.1 mg/dL for women and 0.6–1.2 mg/dL for men.

c Estimated glomerular filtration rate, in mL/min/1.73 m^2^, according to the CKD-EPI creatinine equation (2021).^15^ Normal reference value >90 mL/min/1.73 m^2^. Chronic Kidney Disease if eGFR <60 mL/min/1.73 m^2^.

**Figure 2:**
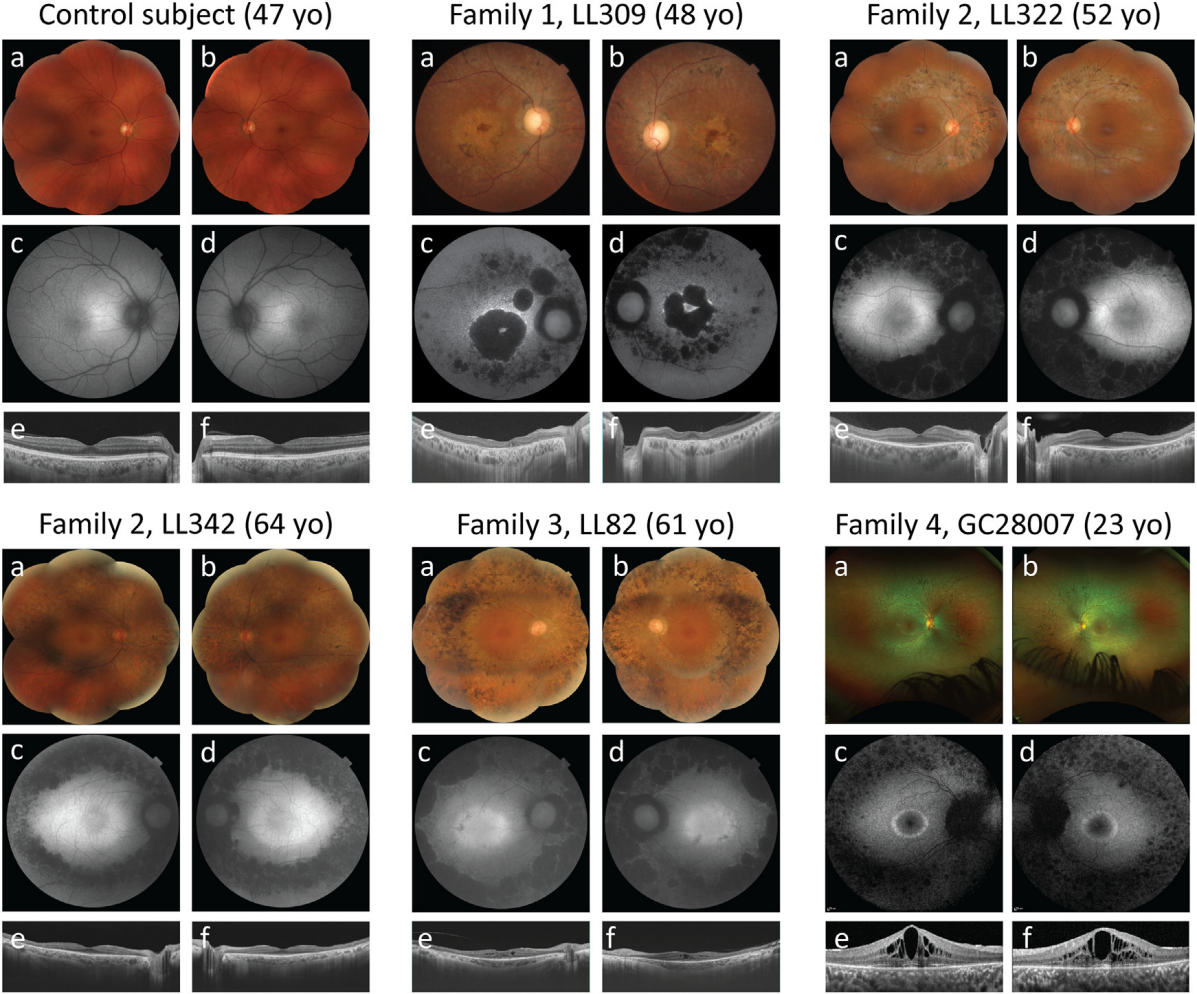
Clinical findings of individuals with bi-allelic variants in *COQ8B*, as well as of a control subject Fundus photographs (a and b), fundus autofluorescence (FAF) (c and d), and optical coherence tomography (OCT) sections (e and f) are shown. For each individual, (a), (c), and (e) refer to the right eye, whereas (b), (d), and (f) refer to the left eye.

Family 1 included one affected individual, LL309. His three siblings and his seven children were all unaffected (Figure 1). Clinically, he first reported photophobia since his teens and central scotomas starting at 40 years of age. A first observation at age 48 revealed RP with early macular involvement (Figure 2; Tables 1 and S1). Genetic analysis revealed the presence of two variants in *COQ8B*, c.1156G>A (p.Asp386Asn) (GenBank: NM_024876.4 and NP_079152.3), referred to as M1, and c.1324G>A (p.Val442Met), referred to as M2 (Figure 1; Table S2). Segregation analysis confirmed compound heterozygosity, since one of his sisters and one of his daughters were heterozygous for M1 and M2, respectively (Figure 1). Aspartate 386 is a very conserved amino acid residue across multiple species, up to budding yeast, residing within the kinase-like domain of COQ8B (Figure 1). The p.Asp386Asn variant is absent from the gnomAD database (v.2.1.1), which lists the genotypes of more than 140,000 control individuals.^16^ Of note, we refrained from using later versions of gnomAD since they may contain genotypes from affected individuals, making frequency-based analysis less reliable. Three different *in silico* tools provided a very high pathogenicity value for this change: MutScore,^17^ VEST4,^18^ and REVEL^19^ (0.851, 0.967, and 0.931 out of 1.00, respectively). The second variant, p.Val442Met, affects an amino acid that is also extremely conserved, again up to budding yeast, and it was present in heterozygously in only two control individuals from the gnomAD dataset (allele frequency = 8.1 × 10^−6^). MutScore, VEST4, and REVEL, all predicted this missense to be deleterious (scores: 0.563, 0.711, and 0.682, respectively).

Family 2 comprised two affected sisters, LL342 and LL322, both diagnosed with RP with relatively late onset of symptoms, in their 30s and 40s (Figures 1 and 2; Tables 1 and S1). They were found to carry two variants, c.1560G>A (p.Trp520Ter), referred to as M3, and c.187C>T (p.Arg63Trp), referred to as M4, in *COQ8B*. As for the previous pedigree, these DNA changes were confirmed to be in *trans* via the analysis of the genotypes of their respective children (Figure 1). M3 is a nonsense variant that results in a premature stop codon in exon 14 of *COQ8B*. Since the variant is located in the last exon of the gene, it is predicted to escape nonsense-mediated mRNA decay (NMD), resulting in a truncated protein with loss of the terminal 25 of 544 amino acid residues.^20^^,^^21^^,^^22^ Based on the three-dimensional model of COQ8A and using the structure of COQ8AΔN255 (PDB: 4PED; residues 255–644) as a reference paralog of COQ8B,^23^ the loss of this C-terminal portion of the protein should not affect its stability or folding, as it is predicted to be a disordered domain (AlphaFold: AF-Q96D53-F1).^24^ However, this region may be important for inter- or intra-molecular protein interactions required for COQ8B allosteric regulation and, therefore, for its function.^24^ M3 was present in gnomAD with a frequency of 7.5 × 10^−5^ with no homozygotes reported. M4, or p.Arg63Trp, is a missense variant involving an amino acid conserved in all vertebrates. Again, MutScore, VEST4, and REVEL, in addition to SIFT,^25^^,^^26^ Polyphen-2,^27^ MutationTaster,^28^ Provean,^29^ and FATHMM,^30^ all predicted it to have deleterious effects on the protein. GnomAD reported a frequency of 3.7 × 10^−3^; importantly, however, two homozygous individuals for this variant were also present.

In family 3, LL82, the only affected individual among a kindred of eight, complained of night blindness since early childhood. Her symptoms worsened during her early 20s, leading to a diagnosis of recessive RP at age 31 (Figure 2; Tables 1 and S1). Similar to the affected individuals from family 2, she harbored the M3 and M4 variants in *COQ8B* in *trans*, as ascertained by segregation analysis conducted in four of her unaffected siblings (Figure 1).

Family 4 included a single affected individual, GC28007 (Figure 1). She was asymptomatic until the age of 23, when she was found to carry signs of retinal dystrophy during a routine optometry test. Following a detailed ophthalmological examination, she was diagnosed with RP in a mild or early form, possibly because of her young age (Figure 2; Tables 1 and S1). At the present time, five years after diagnosis, she reports experiencing mild night vision problems and decreased visual acuity. Genome sequencing with clinical pipeline analysis did not identify any pathogenic genotypes in known IRD genes. Further analysis within the National Genomics Research Library (Genomics England, UK)^31^ revealed the presence of two variants, c.1560G>A (p.Trp520Ter), or M3, and the nonsense c.566G>A (p.Trp189Ter), referred to as M5, inherited in *trans* (Figure 1). This latter variant, which was not found in gnomAD, is predicted to trigger an NMD response since it creates a premature termination codon in exon 7 (out of 14) of *COQ8B* and therefore should result in no protein product.^20^^,^^21^^,^^22^

Although its precise biochemical function has not been fully elucidated, COQ8B is predicted to function as a kinase participating in CoQ biosynthesis, exhibiting ATPase activity in the presence of CoQ intermediates.^32^^,^^33^ Notably, the depletion or inhibition of COQ8B results in a decrease in CoQ production in human cells.^34^ To test the impact of the variants detected in the subjects of this study on COQ8B function, we used NanoBRET target engagement technology, which quantitatively measures the binding ability of a kinase to a ligand, needed to perform its catalytic activity. Specifically, we utilized an expression vector containing a modified luciferase sequence (NanoLuc by Promega, NV2941) fused to the open reading frame of *COQ8B* and used it both as a wild-type expression control and as a template for engineering all the identified variants leading to a viable protein (see supplemental methods and Table S3). We then co-transfected HEK293T cells with assortments of plasmids mimicking the compound heterozygous genotypes detected in affected individuals (e.g., with plasmids containing M1 and M2 in equal amounts, as a proxy for the genotype of LL309). Of note, GC28007’s genotype, including the NMD-insensitive variant M3 in *trans* with the NMD-sensitive variant M5, was mimicked in our assay by transfecting a plasmid carrying M3 only. After 24 h of incubation, cells were supplemented with a serial dilution ranging from 0 to 1 μM of K-10 (N2640), a COQ8B-specific tracer (see supplemental methods). After adding luciferase substrate, the background-corrected luminescence energy transfer was measured.

**Figure 3:**
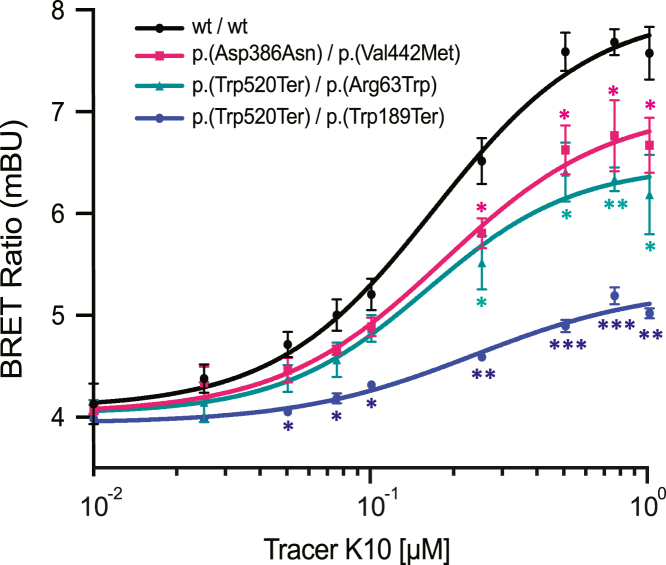
NanoBRET target engagement assay curves Data were obtained following the transfection of HEK293T cells with plasmids carrying the wild-type *COQ8B* cDNA sequence (black) or variants detected in family 1 (pink), families 2 and 3 (green), or family 4 (blue). All points represent the average values of at least 2 technical replicates (range 2–6) for each of 2 biological replicates. Statistical assessment was performed with respect to the wild-type sequence. Error bars indicate standard deviation. ^∗^*p* value < 0.05; ^∗∗^*p* value < 0.01; ^∗∗∗^*p* value < 0.001, by 2-tailed t test. mBU, milli-BRET units.

The NanoBRET resonance energy transfer curves showed statistically significant differences between the binding profile of the control sample, transfected with wild-type plasmids, versus the profiles obtained from cells transfected with plasmids corresponding to the genotypes detected in all the affected individuals, suggesting that the variants identified have a potential deleterious effect on COQ8B function (Figure 3).

We also used this test to assess the specific impact of M4 on COQ8B, due to its relatively high frequency in the general population (∼0.004) and its presence in two homozygous control individuals from gnomAD. To this end, we analyzed the NanoBRET response profile of HEK293T cells transfected with plasmids bearing the M4 change only, mimicking a person homozygous for the p.Arg63Trp missense. Our analysis showed no statistically significant differences between these cells vs. cells transfected with wild-type *COQ8B* plasmids (Figure S1). This indicates that M4 could represent a hypomorphic allele that is disease-causing only when it is in *trans* with a more severe variant (as is the case for individuals LL82, LL322, and LL342), but does not cause disease in the homozygous state. A similar pattern of differential pathogenicity has been recently observed and experimentally validated for other IRD genes, for instance *ABCA4* (MIM: 601691)^35^^,^^36^ and *RP1* (MIM: 603937).^37^ Of note, a literature scan indicated that M4 was previously identified in people with thoracic aortic aneurysm, who also had clear-cut disease-causing variants in genes relevant to this condition. The variant was hypothesized to be a benign modifier for this condition; however, no further data other than statistical association were provided in support of this hypothesis.^38^

Coenzyme Q10 is a lipid-soluble molecule that plays a pivotal role in mitochondrial oxidative phosphorylation, which reduces oxygen to water and produces ATP. In humans, CoQ10 is the most prevalent form of coenzyme Q. It can be both acquired by diet (∼5% of the total amount)^39^ or synthesized endogenously as an endpoint of a series of biochemical reactions,^40^ which can, however, be compromised by the presence of deleterious genetic variants.^3^ The term metabolon designates proteins that form complexes facilitating chains of enzymatic reactions within metabolic pathways.^41^ In the case of eukaryotic metabolons responsible for coenzyme Q biosynthesis, a series of proteins (in humans: COQ3, COQ4, COQ5, COQ6, COQ7, and COQ9)^42^^,^^43^ congregates at the inner mitochondrial membrane to synthesize the final aromatic ring of coenzyme Q. Previous studies have shown that in eukaryotes the orchestration and regulation of the CoQ metabolon relies on COQ8, which in humans is represented by two paralogs: COQ8A and COQ8B.^43^ COQ8B is part of the UbiB protein kinase-like family, which consists of proteins defined by the presence of a protein kinase-like (PKL) domain. Currently, there are no structures available for COQ8B. However, *in silico* analysis predicts a helical domain near the N terminus, followed by an ATP binding cassette (ABC1) transporter domain overlapping with the PKL domain.^44^ Although COQ8 exhibits ATPase activity in the presence of CoQ intermediates,^32^^,^^33^ its role within the CoQ10 biosynthetic pathway remains insufficiently characterized and COQ8B is still considered an orphan kinase. A recent publication, in which the authors analyzed an *in vitro* reconstruction of the entire CoQ metabolon, has shown that COQ8B might function as a COQ3 kinase, a finding supported by intact protein mass spectrometry analysis results.^45^

Deleterious variants in *COQ8B* were first identified in children with SRNS (MIM: 615573)^44^ and later found to be a common cause of this disease, accounting for ∼56% of all instances of SRNS due to CoQ10 deficiency.^46^ Interestingly, a recent review article reported that, out of 140 individuals with bi-allelic variants in *COQ8B* and SRNS, 7 (5%) displayed “retinopathy/ocular abnormalities,”^47^ indicating the presence of rare syndromic manifestations involving both renal and ocular systems. In addition, the M3 variant has been previously identified, in combination with c.1037T>G (p.Ile346Ser), in a 20-year-old Hispanic female presenting with proteinuria, defects in podocyte structure, and abnormal mitochondrial morphology.^24^ It is very clear, however, that all the individuals analyzed in our study have no renal symptoms. All those who underwent nephrological assessment (four out of five) displayed normal kidney function and no detectable proteinuria, the hallmark of podocyte injury. In addition, individuals with COQ8B-associated SRNS have a 74% probability of developing an end-stage kidney disease by the age of 18,^47^ and our subjects are all older than this age. The reasons why some variants lead to SRNS, some to SRNS and ocular phenotypes, and others to non-syndromic IRD are currently not fully understood. There seems to be a weak correlation between truncating and non-truncating variants and the severity of phenotypes, but such an association is not significant for the presence or absence of ocular diseases.^47^ Theoretically, a differential mechanism of pathogenesis related to variants in *COQ8B* could be due to tissue- or organ-specific alternative splicing of the gene, as happens with other similar pathologies.^48^^,^^49^ However, based on the data that are currently available in public databases, differential splicing does not seem to occur for *COQ8B*.^50^^,^^51^ It is also possible that the affected individuals described here suffer from a syndromic disease and would develop renal problems later in their lives. Nonetheless, this hypothesis seems unlikely, since four out of five of our probands are already in their fifth, sixth, or seventh decade of life. On the same note, we cannot rule out that the subjects diagnosed with SNRS could develop retinal symptoms later in their lives.

The association between variants in *COQ8B* and non-syndromic retinal degeneration is poorly understood. Clinical observations have highlighted oxidative stress as a contributing factor to the pathogenesis of retinal diseases.^52^ At the molecular level, oxidative damage is indeed one of the major causes of the death of photoreceptors, especially considering the very high oxygen consumption rates of the retina.^52^^,^^53^ It is therefore reasonable to assume that deficiency of CoQ10, and thus of the mitochondrial respiratory chain, could lead to increased levels of reactive oxygen species and, in turn, to oxidative stress. Alternatively, retinal damage could be linked to a failure to completely restore oxidized cell and photoreceptor disk membranes, as well as lipoproteins in general, which are typical functions of CoQ10. Finally, photoreceptor death could be due to insufficient energy production, again because of a partly deficient respiratory chain. It is known that retinal metabolism requires large quantities of ATP, and therefore photoreceptors may be particularly sensitive to even small reductions in energy production, compared to other cells in the body. Growing evidence supports the premise that CoQ10 plays a protective role for retinal cells, *in vivo* and *in vitro*, indicating a potential exacerbation of retinal disease risk due to the age-related decline of CoQ10 levels.^40^^,^^54^

*COQ8B* expression is ubiquitous although transcriptomics databases suggest a low abundance of transcripts in the retina (https://www.proteinatlas.org/ENSG00000123815-COQ8B/tissue), and specific literature addressing the presence of COQ8B in this tissue is currently missing. Therefore, we assessed COQ8B directly, following protein extraction from HEK293T cells (positive control), a fresh human retina sample, as well as from human retinal organoids.^55^ Western blot analysis confirmed that COQ8B is expressed in the human retina, although in rather low abundance (Figure S2), suggesting that even small variations in CoQ10 synthesis may be highly damaging for this tissue.

Overall, our study highlights the importance of agnostic sequencing investigations of genes previously associated with other diseases and shows the benefits of using an emerging technology such as NanoBRET for orphan proteins, a pipeline that here revealed as well the presence of a potential hypomorphic allele, p.Arg63Trp, in non-syndromic retinal disease.

These findings also open the way to possible therapies based on supplementation of CoQ10, its precursors or its analogs, for individuals carrying disease-causing genotypes in *COQ8B*. A predicted limitation of CoQ10 oral therapy for the treatment of RP lies in this molecule’s inability to effectively penetrate the blood-brain barrier and therefore to reach the retina.^56^ However, CoQ10 or its precursors could be administered via periodical intravitreal injections, as is done for instance with anti-VEGF biopharmaceuticals for the treatment of age-related macular degeneration, which allow the direct perfusion of these molecules to retinal cells.

In summary, this study provides evidence for deleterious variants in *COQ8B* to result in non-syndromic RP. These findings are in agreement with previous data showing that bi-allelic variants in other CoQ10 biosynthesis pathway genes result in retinal dystrophy^9^ and support the hypothesis that ubiquinone has an essential role in preserving normal retinal function.

## Data and code availability

All COQ8B variants identified in this study were submitted to ClinVar (https://www.ncbi.nlm.nih.gov/clinvar/). Their accession numbers are SCV005088525, SCV005088528, SCV005088529, SCV005088530, and SCV005088531.

## Acknowledgments

We would like to acknowledge all affected individuals and their families for taking part in this study, and The IOB Human Organoid Platform, especially Larissa Utz and Pierre Balmer. We are also grateful to Temurkhan Ayupov, Ilaria Gregorio, Verónica Moreno, and Álvaro Herrero for their technical support and to Sitta Föhr for reviewing this manuscript.

Funding was received from the following agencies: The 10.13039/501100001711Swiss National Science Foundation (grant # 204285 to C.R.), Fight for Sight UK (Early Career Investigator Award [grant no. 5045/46 to G.A.]), and 10.13039/501100017645Moorfields Eye Charity (Stephen and Elizabeth Archer in memory of Marion Woods) (G.A.). This research was supported by the 10.13039/501100000272National Institute for Health and Care Research (NIHR) Biomedical Research Center at Moorfields Eye Hospital NHS Foundation Trust and the UCL Institute of Ophthalmology, United Kingdom.

This research was made possible through access to data in the National Genomic Research Library, which is managed by Genomics England Limited (a wholly owned company of the Department of Health and Social Care). The National Genomic Research Library holds data provided by affected individuals and collected by the NHS as part of their care and data collected as part of their participation in research. The National Genomic Research Library is funded by the 10.13039/501100000272National Institute for Health Research and NHS England. The 10.13039/100010269Wellcome Trust, 10.13039/501100000289Cancer Research UK and the 10.13039/501100000265Medical Research Council have also funded research infrastructure.

BioRender.com was used to create some parts of the graphical abstract.

## Declaration of interests

The authors declare no competing interests.

## Web resources

GenBank, https://www.ncbi.nlm.nih.gov/genbank/
OMIM, https://www.omim.org/
RCSB PDB, https://www.rcsb.org/

## Supplemental information

Document S1. Figures S1 and S2 and supplemental methods

Table S1. Additional ophthalmic features of the individuals analyzed

Table S2. DNA variants identified and their features

Table S3. Primers used in this study

Document S2. Article plus supplemental information

## Footnotes

Supplemental information can be found online at https://doi.org/10.1016/j.ajhg.2024.08.005.
