# Supplementary material for: Bi-allelic variants in COQ8B, a gene involved in the biosynthesis of coenzyme Q10, lead to non-syndromic retinitis pigmentosa
Source: Am J Hum Genet. 2024 Sep 2;111(10):2299–306. doi: 10.1016/j.ajhg.2024.08.005 (PMC11480794; doi:10.1016/j.ajhg.2024.08.005)
Supplement: Document S1. Figures S1 and S2 and supplemental methods [file mmc1.pdf]

The American Journal of Human Genetics, Volume *111*

Supplemental information

Bi-allelic variants in *COQ8B*, a gene involved in the biosynthesis of coenzyme Q10, lead to non-syndromic retinitis pigmentosa

Ana Belén Iglesias-Romero, Karolina Kaminska, Mathieu Quinodoz, Marc Folcher, Siying Lin, Gavin Arno, Joaquim Calado, Andrew R. Webster, Alexandre Moulin, Ana Berta Sousa, Luisa Coutinho-Santos, Cristina Santos, and Carlo Rivolta

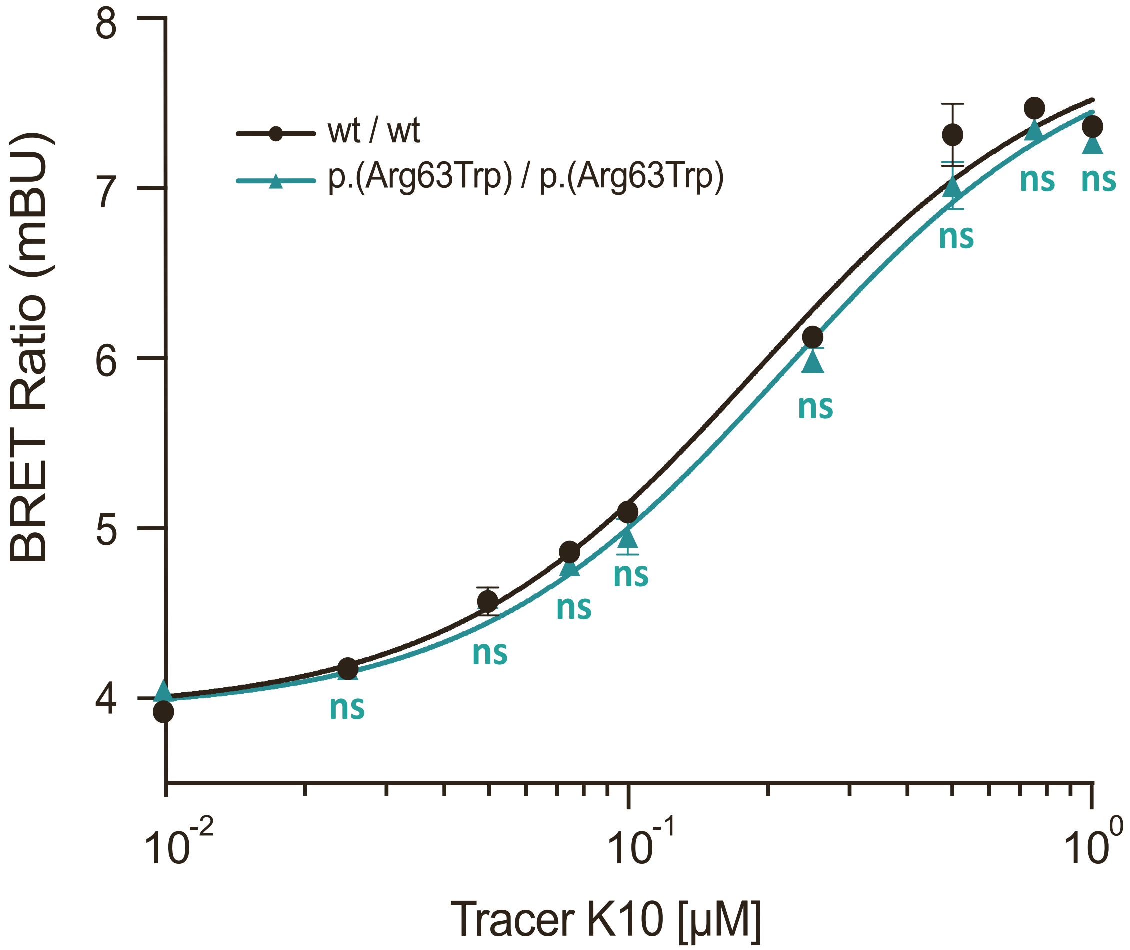

Figure S1. NanoBRET target engagement analysis of wt vs. p.(Arg63Trp) homozygous. BRET ratios observed with Tracer K10 in COQ8B wt and genotype corresponding with variant p.(Arg63Trp) in homozygosity. All points represent the average values of at least 2 technical replicates (range 2–6) for each of 2 biological replicates. Statistical assessment was performed with respect to the wild-type sequence. Error bars indicate standard deviation. mBU: milli-BRET units. ns: not significant.

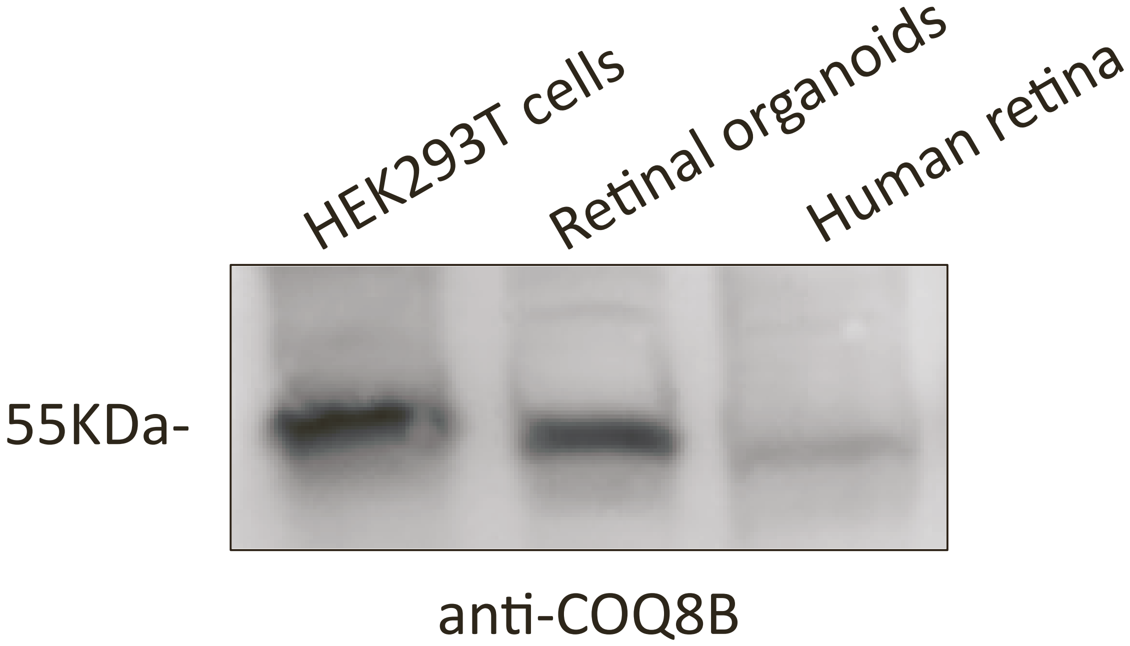

Figure S2. Western blot analysis of COQ8B protein in HEK293T cells, human retinal organoids (week 26) and human retina. 50ug of total protein extract were loaded per well, in all lanes.

Supplementary Methods

Ophthalmological assessment

All patients underwent a complete and standardized ophthalmological evaluation, including assessments of best corrected visual acuity (BCVA), slit-lamp examination, fundus imaging (center and periphery, with autofluorescence), optical coherence tomography (OCT), electroretinography (ERG), color vision tests, and visual field assessments (Goldmann or Humphrey). On this basis, a clinical diagnosis was made at the time of enrollment. DNA was obtained from whole-blood or saliva samples. Cohorts were assembled based on their primary center of care, in Portugal and in the UK. The origin and the ethnicity of all participating individuals were self-reported.

Nephrological assessment

Patients were evaluated by nephrology consultants with expertise in inherited kidney phenotypes. Protein-to-creatinine ratio in random spot urine and the CKD-EPI creatinine equation (2021)[1] were used, respectively, for proteinuria screening and the estimation of glomerular filtration rate (eGFR). Four out of five patients underwent kidney ultrasound evaluation.

Expression vector and site-directed mutagenesis

The Q5 Site-Directed Mutagenesis Kit (New England Biolabs) was used following the manufacturer’s instructions. Clones were confirmed by direct Sanger sequencing in Mycrosynth (Switzerland). Oligos used in this study are listed in Table S3.

Cell culture and transfection

HEK293T cells were cultured in Dulbecco's Modified Eagle Medium, (DMEM, 4.5 g/L glucose, 110 mg/ml sodium pyruvate, Gibco, 10569-010) and 10% fetal bovine serum (Sigma, F7524). All cells were grown at 37 °C in a humidified incubator maintained at 5% CO_2.

NanoBRET TE Intracellular Kinase Assay

The NanoBRET target engagement (TE) COQ8B assay kit was purchased from Promega (Promega, N2640) and used according to the manufacturer's instructions as follows: HEK293T cells were reverse transfected as described above with the NanoLuc-COQ8B Fusion Vector (Promega, NV2941) containing the wild type sequence or one of the four mutant variants. 500µl of Opti-MEM containing FuGENE HD transfection reagent DNA-DNA carrier complex (15 µL FuGENE, 0.5 µg NanoLuc-COQ8B Fusion Vector, and 4.5 µg DNA carrier) were briefly incubated at room temperature (RT) and mixed with 10 mL of HEK293T cells in suspension (2×10^5 cells/mL). Cells were dispensed into Corning 96-well, white, solid bottom, medium binding assay plates (Corning, CLS3917) as 100 µl/well and incubated for 24 hours at 37°C and 5% CO_2. 5µL of 20X NanoBRET Tracer Reagent K-10 activated with Tracer Dilution Buffer (Promega) at a 1:4 ratio were added in increasing concentrations, as per manufacturer's instructions. Following a brief stir to ensure reagent mixing, the assay plates were incubated at 37°C and 5% CO_2 for 2 hours before equilibration to RT for 15 minutes. 50 µL of 3X Complete Substrate plus Inhibitor Solution (1:166 dilution of NanoBRET Nano-Glo Substrate plus 1:500 dilution of Extracellular NanoLuc Inhibitor in phenol red-free OptiMEM assay medium) were added. The filtered luminescence was then measured using GloMax Discover microplate

reader (Promega) equipped with a 450nm (8-nm band pass) filter (donor) and a 600nm long pass filter (acceptor). BRET was calculated by dividing the acceptor emission by the donor emission. The values were background corrected. Data was plotted in GraphPad Prism software using the agonist versus response-variable slope fitting.

Preparation of human retina and western blotting

Human retina proteins were electrophoretically separated on 10% SDS-polyacrylamide gels and transferred to polyvinylidene difluoride (PVDF) membranes. The membranes were then incubated for 24 hours at 4 °C with the primary antibody (Invitrogen, PA5-55551). The appropriate secondary antibody conjugated to horseradish peroxidase (Invitrogen) was applied to the membrane. Reactions were developed with chemiluminescence substrate (Thermo Fisher, 34580). Images were taken with a gel documentation system (ImageQuant 800, Cytiva).

Exome sequencing (ES), genome sequencing (GS) and data processing

ES was performed at CeGaT GmbH (Tübingen, Germany) for LL309 and LL322, and at the Institute of Genomics of the University of Tartu (Estonia) for LL82. There, sequencing libraries were generated using the Twist Human Core Exome Plus kit (Twist Bioscience, CeGaT) or the Nextera Rapid Capture Exome kit (Illumina, Institute of Genomics, Tartu), following manufacturers' protocols. Libraries underwent paired-end sequencing on a Novaseq 6000 (CeGaT) or a HiSeq2500 (Institute of Genomics, Tartu) platform (Illumina) resulting in sequences of 100 or 150 bases. The total output per sample was of at least 10 Gbases, representing an average coverage of >120X in targeted regions and resulting in ~90% of the

targeted regions with a coverage higher than 20X. The processing of the sequencing data (mapping, variant calling, and variant annotation) was performed as described previously.[2] For patient GC28007, the DNA sample was sent for GS at the UK NHS Genomic Medicine Service and performed at the national GS provider (Illumina). Analyses and interpretations of the results were conducted at the North Thames Genomic Laboratory Hub (GLH), which is based at Ormond Street Hospital.

Targeted Sanger sequencing

Primer3Plus was used to design primers for polymerase chain reactions (PCR), performed using the GoTaq polymerase (Promega) and 2ng of template DNA, according to the manufacturer's protocol. All PCR products were treated with ExoSAP-IT (Thermo Fisher) and Sanger sequencing was performed by Microsynth (Switzerland). Sequences were visualized and compared to the gene's reference sequence (Ensembl, GRCh37) with the CLC Genomics Workbench 12 software (QIAGEN). Oligos used are listed in Table S3.

Statistical analysis

GraphPad Prism software was used to analyze and plot the results of the NanoBRET target engagement assays. The concentration of the agonist versus response-variable slope fitting was used. For all cases, two biological replicas were performed, including a minimum of two technical replicas per tracer concentration and genotype. An unpaired t-test, bilateral with equal variance, was used to compare the wild-type with the patients identified in this study.

REFERENCES

1. Inker, L.A., Eneanya, N.D., Coresh, J., Tighiouart, H., Wang, D., Sang, Y., Crews, D.C., Doria, A., Estrella, M.M., Froissart, M., et al. (2021). New Creatinine- and Cystatin C-Based Equations to Estimate GFR without Race. N Engl J Med 385, 1737-1749.
2. Peter, V.G., Kaminska, K., Santos, C., Quinodoz, M., Cancellieri, F., Cisarova, K., Pescini Gobert, R., Rodrigues, R., Custodio, S., Paris, L.P., et al. (2023). The first genetic landscape of inherited retinal dystrophies in Portuguese patients identifies recurrent homozygous mutations as a frequent cause of pathogenesis. PNAS Nexus 2, pgad043.
